# Health-related quality of life in different clinical subgroups with typical AFL who have undergone cavo-tricuspid isthmus ablation

**DOI:** 10.1186/1477-7525-10-90

**Published:** 2012-08-06

**Authors:** Javier García Seara, Francisco Gude, Pilar Cabanas, José L Martínez-Sande, Xesús Fernández López, Antonio Hernández Madrid, Concepción Moro, José R González Juanatey

**Affiliations:** 1 Cardiology Department, Hospital Clinico de Santiago de Compostela, Calle Choupana s/n, Santiago de Compostela 15703, Spain; 2 Epidemiology Department, Hospital Clinico de Santiago de Compostela, Calle Choupana s/n, Santiago de Compostela, 15703, Spain; 3 Cardiology Department, Hospital Ramón y Cajal, Carretera de Colmenar Viejo km 9.1, Madrid, 28034, Spain

**Keywords:** Atrial flutter, Cavotricuspid isthmus ablation, Health-related quality of life

## Abstract

**Background:**

To evaluate changes in health-related quality of life (HRQOL) in different sub-groups of a cohort of patients with typical atrial flutter (AFL) treated with cavotricuspid isthmus (CTI) radiofrequency catheter ablation.

**Methods:**

95 consecutive patients due to undergo CTI ablation were enrolled in a study involving their completion of two SF-36 HRQOL questionnaires, before ablation and at one-year follow-up**.**

**Results:**

88 of the initial 95 patients finished the study. Regardless of whether patients experienced atrial fibrillation (AF) during follow-up, a statistically significant improvement in HRQOL was observed, compared with pre-ablation scores and in all dimensions except Bodily Pain. However, patients without AF during follow-up had significantly higher absolute HRQOL scores in most dimensions. No differences were seen in most HRQOL dimensions, with respect to AFL type (paroxysmal, persistent) or duration, whether AFL was first-episode or recurrent, Class I-III drug dependent, sex, or presence of structural heart disease or tachycardiomyopathy. Patients with persistent AFL showed the greatest improvement in HRQOL when they also had a ventricular cycle length ≤500 ms. The combination of recurrent AFL, ventricular cycle length ≤500 ms and structural heart disease led to a significantly greater improvement in physical HRQOL dimensions than did first-episode AFL, no structural heart disease and ventricular cycle >500 ms. The only independent factor associated with a greater improvement was structural cardiopathy.

**Conclusions:**

CTI-ablation treatment leads to a significant improvement in HRQOL in patients with typical AFL. Patients with AF during follow-up show a significantly lower HRQOL at one-year post-ablation. The only independent risk factor found to be associated with a greater improvement in the physical summary component was structural cardiopathy.

## Background

Cavotricuspid isthmus (CTI) ablation is a first-line treatment in recurrent typical atrial flutter (AFL). It is strongly indicated for patients with poor clinical tolerance or in whom AFL is a sequela of treatment for atrial fibrillation (AF) with Class I or III drugs [1-4]. Previous studies have described a reduction in symptoms and improvement in quality of life in AFL patients following CTI ablation [5-8]. These studies have used the US population as their reference population, but have not adjusted their data for age or sex, factors which significantly affect health-related quality of life (HRQOL). We have recently reported the minimal clinically important differences which give rise to changes in HRQOL in CTI ablation patients, thus providing a conceptual basis for distinguishing whether changes in HRQOL attributable to treatment are perceptable to the patient [9].

None of these studies, however, have analysed differences in HRQOL in AFL patients with respect to age, sex, AFL sub-type (paroxysmal or persistent) or duration, whether AFL is a first episode or recurrence, presence or absence of AF, tachycardiomyopathy, anticoagulant treatment, structural heart disease, or ventricular cycle length. The aim of our study, therefore, was to determine changes in HRQOL in several sub-groups of a cohort of CTI-dependent AFL patients, before CTI radiofrequency catheter ablation (basal) and at one-year follow-up.

## Patients and methods

### Patient population

95 patients were referred sequentially to our Cardiac Interventional Electrophysiology Laboratory at the University of Santiago de Compostela Hospital, between January 2003 and March 2005, who fulfilled all the following criteria, were enrolled: a.) over 18 yrs of age; b.) had at least one AFL episode in the preceding 6 months, shown by 12-lead ECG; c.) history of lone AFL or predominant AFL if presenting with concomitant AF, or AFL resulting from anti-AF treatment with Class I or III drugs; d.) electrophysiological confirmation of CTI AFL or CTI permeability if the procedure was carried out in sinus rhythm. In the latter, the ECG of the clinical episode had to indicate common typical AFL.

Exclusion criteria were: a.) no CTI-dependent AFL; b.) cardiac surgery or other cardiac intervention (e.g. coronary angioplasty or pacemaker implantation) in the past 30 days; c.) carrier of implantable cardioverter defibrillator; d.) life expectancy less than one year; or e.) incomplete HRQOL questionnaire.

### Ablation procedure

All patients had fasted for at least 6 hr and stopped taking oral anticoagulants at least two days previous to electrophysiological procedure. A standard quadripolar catheter (Usci-Bard Inc) was used to map the bundle of His, a 10-pole catheter (Usci-Bard Inc) to locate the coronary sinus, and a 12-pole Halo XP catheter (Cordis-Webster Inc) was used to record activation in the anterolateral aspect of the right atrium. An 8 mm-tip catheter was used for radiofrequency (RF) ablation. RF was delivered for 60 s at each location. The maximum power output was 90 watts, and maximum temperature was 55°C. CTI-dependence was confirmed by concealed entrainment if AFL rhythm was either present at the beginning of the electrophysiological analysis or induced in the laboratory. If the patient was in sinus rhythm bidirectional CTI permeability was determined before ablation.

The objective of the ablation procedure was to effect a bidirectional conduction block across the CTI [10,11]. Bidirectional block was determined by the sequence of electrical activity between the right atrium, bundle of His and coronary sinus following a 600 ms stimulation in the coronary sinus and in the inferolateral wall of the right atrium. Persistence of bidirectional CTI conduction block was tested at 20 min after procedure.

### HRQOL questionnaire

The Spanish version of the Short-Form-36 (SF-36) HRQOL questionnaire [12-14] was filled in by all enrolled patients before CTI ablation treatment and at one-year follow-up. This questionnaire consists of 8 scales or dimensions, each containing two or more items which in turn have a possible score of 0 to 100 (100 signifying a healthy state) [13]. By combining scores for each dimension, two summary scores are derived: the physical component summary (PCS) and the mental component summary (MCS) [14].

### One-year follow-up

All enrolled patients were booked into clinics for follow-up at 3, 6 and 12 months post-ablation. Any visit to the Cardiology Department or to the Accident and Emergency Service was registered in the electronic clinical database. At 6 months post-ablation, any asymptomatic abnormalities were recorded using an ambulatory Holter device worn for a period of 7 days.

### Statistical analysis

Results of the SF-36 questionnaires were analysed using Student’s t-test and Mann–Whitney non-parametric tests, as appropriate. Normality of distribution and homogeneity of variance were assessed by Kolmogorov-Smirnov and Levene tests, respectively. Comparisons of the results of the SF-36 questionnaires before ablation (basal) and at one-year follow-up were made using Wilcoxon test for paired data. Differences between basal and one-year follow-up component summaries were calculated. These covariates were categorized into two groups: i) improvement or ii) worsening. To investigate which covariates were associated with improvement in mental and physical component summaries, two logistic regression models were performed. P < 0.05 was considered significant.

### Ethical considerations

The study was carried out in accordance with the Principles of the Declaration of Helsinki (1975) and approved by the Ethical Committee of Clinical Investigation in Galicia. All enrolled patients gave their written informed consent.

## Results

### Patient characteristics

Of 104 patients with AFL referred sequentially to our Electrophysiology Laboratory, 95 had typical (CTI-dependent) AFL and were enrolled in the study.

Table1 shows AFL-related and other characteristics for the patient population before CTI-ablation treatment. The most common type of AFL was paroxysmal (56%). Almost 40% of patients had previously received electrical or pharmacological cardioversion, 43% had concommitant AF, and 44% of patients were undergoing CTI ablation for their first episode of AFL. There was a 17% incidence of tachycardiomyopathy, and only 58% of patients had been on anticoagulant medication. 15% of patients had Class I or III drug-related AFL, with the majority of cases being attributable to amiodarone.

**Table 1:** Basal characteristics of enrolled AFL patients (before CTI-ablation treatment; n = 95)

Age (yr)	64.4 ± 10.6
Males, n (%)	77 (81,1)
COPD, n (%)	20 (21,1)
Hypertension, n (%)	47 (49,5)
Diabetes mellitus, n (%)	19 (20,0)
Obese, n (%)	25 (26,3)
LVEF <50%, n (%)	22 (23,1)
Hypertensive cardiopathy, n (%)	37 (38,9)
Valvulopathy, n (%)	19 (20,0)
Ischaemic heart disease, n (%)	14 (14,7)
Heart Failure, n (%)	18 (18,9)
Dilated cardiomyopathy, n (%)	21 (22,1)
Previous cardiac surgery, n (%)	12 (12,6)
No heart disease, n (%)	20 (21,1)
Cor Pulmonale, n (%)	5 (5,3)
Type of AFL, n (%)	
Persistent	53 (55,8)
Paroxysmal	42 (44,2)
Ventricular cycle length, ms	653 ± 223
Paroxysmal	641 ± 260
Persistent	686 ± 192
Duration of AFL, months	12 [3, 48]
AFL episodes, n	2 [1,7]
Previous ECV, n (%)	22 (23,2)
Previous PCV, n (%)	15 (15,8)
Previous anticoagulation treatment, n (%)	55 (57,9)
1 year follow –up anticoagulation treatment n (%)	63 (66,3)
Previous AF, n (%)	41 (43,2)
Class I- III drugs related AFL, n (%)	14 (14,7)
Flecainide	3 (3,1)
Amiodarone	11 (11,6)
Tachycardiomyopathy, n (%)	16 (16,8)

### One-year follow-up

Acute success was achieved in all patients. No patient died in hospital, but 6 died during the follow-up period and from the following causes: lung cancer, severe aortic stenosis, respiratory insufficiency due to COPD, respiratory infection and sudden death outside the hospital (2 cases, of which one was due to a pulmonary embolism). One patient was unable to complete the HRQOL questionnaire due to a cerebrovascular accident with neurological sequelae. Thus, 88 patients successfully completed follow-up and their questionnaires.

13 patients had recurrences of typical AFL (14.6%) before the end of the follow-up period, of whom 12 underwent a repeat CTI ablation and the other patient was treated by electrical cardioversion. 24 patients (25%) experienced episodes of AF during follow-up, of whom 20 (83%) had previously had AF, and compared with a total of 41 (43%) having had AF before ablation treatment.

### Quality of life

#### Global

**Figure 1:**
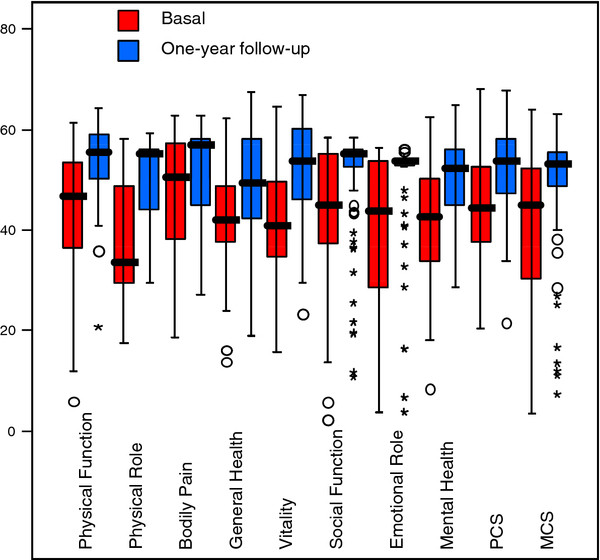
**Box plot depicting HRQOL scores before CTI-ablation treatment (basal) and at one-year follow-up in the whole cohort of AFL patients (n = 88), for all HRQOL dimensions.** Horizontal bars indicate median. PCS: Physical Component Summary; MCS: Mental Component Summary.

A significant improvement in HRQOL was seen at one year, compared with basal values and with respect to all dimensions in the SF-36 questionnaire (p < 0.05 all dimensions; Figure1).

#### Socio-demographic variables (age and sex)

No significant differences in HRQOL at one year, compared with basal scores, were found in relation to patient age. Women showed a significant improvement in the Emotional Role compared with men. No other gender-dependent HRQOL differences were recorded at one year. Neither were any clinical differences observed in outcome or complications of CTI ablation between men and women.

#### AFL characteristics

No differences in HRQOL at one year compared with basal values were seen with respect to AFL type (paroxysmal or persistent), AFL duration, ventricular cycle length or presence of tachycardiomyopathy. In terms of number of AFL episodes (first episode or recurrent) a significant improvement was seen with respect to the Physical Function dimension in patients with recurrent compared with first-episode AFL (Table2a). Patients with structural heart disease showed a significant improvement at one year in terms of the Physical Role dimension with respect to no structural heart disease group (Table2a). Patients with Class I drug-related AFL reported similar changes in HRQOL to all other AFL patients, except in terms of the two dimensions of General Health and Bodily Pain in which the improvements they reported were significantly less.

**Table 2:** Differences in HRQOL at one-year follow-up after CTI-ablation treatment compared with basal scores in different sub-groups of AFL patients

A. Differences in HRQOL with respect to presence of structural heart disease and number of AFL episodes. Data are expressed as mean ± SD; *p < 0.05. AFL: Atrial flutter						
	**Structural heart disease**			**N° of AFL Episodes**		
	**Yes**	**No**	**p**	**1 episode**	**> 1episode**	**p**
	**(n = 68)**	**(n = 20)**		**(n = 37)**	**(n = 51)**	
Physical Function	26.5 ± 20.8	17.5 ± 20.9	0.10	18.4 ± 17.5	28.8 ± 22.4	0.02*
Role Physical	49.8 ± 46.9	28.7 ± 37.4	0.04*	40.5 ± 40.1	48.3 ± 49.4	0.41
Bodyly Pain	8.6 ± 30.5	14.0 ± 36.5	0.55	9.1 ± 30.5	10.3 ± 33.1	0.85
General Health	16.1 ± 20.3	12.6 ± 15.9	0.42	13.2 ± 22.8	16.9 ± 16.5	0.40
Vitality	27.4 ± 23.8	16.7 ± 23.7	0.08	24.3 ± 21.7	25.5 ± 25.8	0.81
Social Function	14.8 ± 25.6	22.1 ± 23.3	0.24	18.4 ± 26.1	15.2 ± 24.5	0.56
Role Emotional	29.9 ± 39.5	36.7 ± 44.5	0.54	30.6 ± 38.0	32.0 ± 42.7	0.87
Mental Health	18.5 ± 23.7	15.4 ± 21.4	0.57	16.5 ± 23.0	18.7 ± 23.3	0.66
B. Differences in HRQOL in persistent AFL with respect to ventricular cycle length. Data are expressed as mean ± SD; *p < 0.05; **p < 0.01						
		**Ventricular cycle length**				
		**≤500 ms (n = 10)**		**>500 ms (n = 30)**		**p**
Physical Function		32.5 ± 30.9		21.2 ± 16.5		0.17
Role Physical		71.9 ± 36.4		36.6 ± 44.3		0.04*
Bodily Pain		20.5 ± 36.2		5.5 ± 27.1		0.20
General Health		34.3 ± 15.0		11.0 ± 22.4		0.009**
Vitality		45.6 ± 27.3		24.3 ± 23.9		0.04*
Social Function		26.6 ± 33.7		18.9 ± 25.8		0.40
Role Emotional		50.0 ± 39.8		29.8 ± 39.9		0.21
Mental Health		35.5 ± 21.4		16.6 ± 21.5		0.03*
C. Differences in HRQOL with respect to combined characteristics. Data are expressed as mean ± SD; *p < 0.05. PCS: Physical Component Summary; MCS: Mental Component Summary; AFL: atrial flutter						
		**Recurrent AFL + ≤500 ms cycle + structural heart disease (n = 12)**		**1st episode AFL + >500 ms cycle + no structural heart disease (n = 4)**		**p**
Physical Function		38.7 ± 24.1		12.5 ± 17.1		0.03
Physical Role		60.4 ± 40.5		0.0 ± 0.0		0.02*
Bodily Pain		6.3 ± 28.8		−6.5 ± 37.2		0.57
General Health		24.1 ± 20.5		10.5 ± 17.0		0.41
Vitality		31.2 ± 28.0		16.2 ± 26.2		0.34
Social Function		21.9 ± 28.2		23.1 ± 21.7		0.75
Emotional Role		33.3 ± 44.9		41.7 ± 50.0		0.94
Mental Health		21.3 ± 24.6		23.0 ± 18.3		0.94
PCS		35.4 ± 27.7		−8.2 ± 22.2		0.02*
MCS		20.2 ± 38.3		40.4 ± 38.3		0.41

Within the persistent AFL group, those with a ventricular cycle ≤500 ms showed a greater improvement in HRQOL in all dimensions and which reached significance in Physical Role, General Health, Vitality and Mental Health (Table2b).

In terms of the dimensions Physical Function, Physical Role and PCS, the sub-group of patients with recurrent AFL, structural heart disease and a ventricular cycle length ≤500 ms showed a significantly greater improvement than first-episode AFL patients without structural heart disease but with a ventricular cycle >500 ms; (Table2c).

#### Atrial fibrillation

Patients with a history of AF gave lower basal scores in most dimensions of the HRQOL questionnaire than those with no previous AF, although these differences were not statistically significant except in General Health dimension (Table3). Both AF-in-follow-up and non-AF-in-follow-up groups recorded significant improvements in all but one dimension (Bodily Pain) at one year with respect to basal scores, and several of these reached p < 0.001. There was no statistically significant difference between the two groups (Table3). Absolute HRQOL scores at one year, however, were significantly higher for patients who had not experienced AF during follow-up than for those who had, in all dimensions except Physical Role, Bodily Pain and Social Function (Table3).

**Table 3:** Differences in HRQOL with respect to the presence of AF in Follow-up

	**AF in Follow-up**		**No AF in Follow-up**	
	**(n = 23)**		**(n = 65)**	
	**Basal**	**1-year FU**	**Basal**	**1- year FU**
Physical Function [ns]	56.7 ± 23.2	83.0 ± 14.2♦♦	61.9 ± 23.8	86.1 ± 15.8♦♦
Physical Role [**]	23.9 ± 36.5	60.4 ± 39.2♦♦	34.1 ± 42.2	82.3 ± 30.8♦♦
Bodily Pain [ns]	57.5 ± 33.9	69.7 ± 30.0	72.7 ± 30.7	81.8 ± 27.4
General Health [**]	34.7 ± 20.1	46.1 ± 21.3♦	45.8 ± 17.2♠	62.8 ± 18.9♦
Vitality [*]	36.7 ± 23,8	58.9 ± 21.1♦	44.7 ± 24.4	71.0 ± 23.2♦♦
Social Function [ns]	67.2 ± 29.8	83.1 ± 26.3♦♦	75.3 ± 28.2	91.7 ± 18.4♦♦
Emotional Role [*]	43.5 ± 45.4	76.8 ± 41.9♦♦	62.1 ± 42.9	92.8 ± 21.6♦♦
Mental Health [**]	52.0 ± 24.4	64.9 ± 18.7♦	57.3 ± 23.0	76.7 ± 17.4♦♦
PCS [*]	42.1 ± 27.0	62.9 ± 22.9♦♦	50.6 ± 27.3	75.0 ± 26.9♦♦
MCS [*]	47.8 ± 34.8	67.3 ± 32.8♦♦	59.1 ± 33.1	79.7 ± 20.9♦♦

HRQOL scores at basal and at one-year follow-up in patients with and without AF during follow-up. Data are expressed as mean ± SD. PCS: Physical Component Summary; MCS: Mental Component Summary; AF: atrial fibrillation. FU: Follow-up.

[*] p < 0.05, [**] p < 0.01, [ns] no significant, for the differences in HRQOL at one-year follow-up between patients with or without AF during follow-up.

♦ p < 0.05; ♦♦ p < 0.01: p-values for the differences at one-year follow-up compared with basal values in each group.

♠ p = 0.02 for the differences at basal between patients with or without AF during follow-up. No significant differences in any of HRQOL remaining dimensions at basal between the two groups.

No significant difference were found for HRQOL differences values (one year follow-up - basal) between patients with or without AF during follow-up.

#### Anticoagulation treatment

Pre-ablation HRQOL scores were similar regardless of whether patients were taking basal anti-coagulant medication. At one-year follow-up, a significant difference was observed between these two patient groups with respect to one dimension only, namely that of General Health which was higher in the group not receiving oral anti-coagulation treatment.

After adjusting for potential confounders, the only factor found to be associated with a greater improvement in the physical component (PCS) was the presence of structural cardiopathy. No other variable was found to be associated with improvement in the summary components at one-year follow-up (Table4).

**Table 4:** Associated factors with improvement in physical and mental component summaries at one-year follow-up

	**PCS**		**MCS**	
	**OR (95% CI)**	**p-value**	**OR (95% CI)**	**p-value**
Age, yr	0.95 (0.98, 1.02)	0.126	1.03 (0.97, 1,09)	0.337
Gender	1.37 (0.25, 7.37)	0.714	0.93 (0.19, 4.48)	0.923
Structural heart disease	5.83 (1.14, 29.7)	0.034	1.58 (0.36, 6.92)	0.545
AF at follow-up	2.96 (0.38, 22.9)	0.298	1.49 (0.34, 6.55)	0.596
AFL recurrence	0.29 (0.05, 1.77)	0.178	5.12 (0.51, 51.2)	0.164
> 1 episode of AFL	1.66 (0.45, 6.14)	0.447	0.34 (0.11, 1.11)	0.075
Ventricular cycle length	1.51 (0.36, 6.30)	0.573	1.60 (0.49, 5.28)	0.439

PCS means physical component summary; MCS, mental component summary; AF, atrial fibrillation; AFL, atrial flutter; OR, odds ratio; 95%CI, 95% confidence interval.

Codes used in the logistic regression analysis: Gender (0, female; 1, male). Structural heart disease (0, no; 1, yes). AF at follow-up (0, no; 1, yes). AFL recurrence (0, no; 1, yes). > 1 episode of AFL (0, no; 1, yes). Ventricular cycle length (0, > 500 ms; 1, < 500 ms).

## Discussion

The aim of the current study was to identify demographic or clinical parameters associated with the greatest improvement of HRQOL scores in patients with typical AFL undergoing CTI catheter ablation. Thus we have analysed, for the first time, changes in HRQOL scores before ablation and at one-year follow-up, in terms of several patient sub-groups. Taken as a whole, the population of typical AFL patients that we studied showed a clinically significant improvement in HRQOL one year after undergoing CTI ablation [9]. This result was notable given that the cohort included patients with concomitant AF (43%) and also only a single episode of AFL (44%). After adjusting for age, sex, and other cardiovascular-related parameters, the presence of structural cardiopathy was associated with an improvement in the physical summary component that was 5.8 greater than that of the absence of structural cardiopathy, and the only variable that made a difference that was statistically significant.

The recurrence rate of AFL at one year was 14.6%, a slightly higher level than reported for the majority of other patient series (20% at 1 year, [5,15] 10%[16] and 6.3% at 16 meses, [17] 4.8% at 21 meses [18]). One potential partial explanation would be that while a 8–mm- tip catheter was used in 93 patients (97.9%), an irrigated-tip catheter was used in the remaining two patients (2.1%), since it is known that an irrigated tip produces more profound lesions than an 8- mm- tip catheter. However, to date no associated differences in outcome have been demonstrated either acutely [16,19] or in the longer term [17] after treatment of CTI-dependent AFL with an irrigated-tip or 8-mm- tip catheter. We therefore conclude that our increased recurrence levels were probably due to factors other than catheter tip, and most likely related to CTI morphology.’

Patients who developed AF during follow-up also showed a significant improvement in HRQOL at one year, except in the dimension of Bodily Pain. Although the group who developed AF during follow-up showed a smaller improvement in HRQOL with respect to the group that did not develop AF, the difference between the two groups did not approach significance in any dimension. Without further analysis, this result contrasts with that of Lee et al [8] in which a history of AF was the only factor associated with reduced improvement of HRQOL in patients with typical AFL undergoing CTI ablation. However, when we took into account HRQOL at one-year follow-up we also found in our cohort that for most dimensions final scores were significantly better in patients who had not experienced AF during follow-up than in those who had. These data suggest that the differences seen in basal HRQOL (lower in patients with a history of AF) maintained and formed a component of the scores obtained at one year after CTI catheter ablation (smaller improvement in patients experiencing AF during follow-up), thus accounting for the differences seen at one year in most of dimensions approaching or reaching significance. Interpreted thus, our results for HRQOL at one-year follow-up agree more closely with those of Lee et al [8].

A long-term (27 months) benefit in quality of life in patients undergoing CTI ablation with a history of AF has previously been reported by Anselme [20]. Those results are similar to ours with respect to the 8 dimensions of the SF36 questionnaire, showing a significant improvement in all items except for Bodily Pain. In that study, the improvement was smaller in patients with a history of AF than in those without, but the difference between those two groups was not significant. However, in our cohort, basal scores were significantly lower overall, and especially in patients who had presented AF during follow-up. In Anselme’s cohort, moreover, patients with a history of AF also had higher basal scores than those without, in the majority of items.

Whilst the presence of AF reduced the benefit of CTI ablation in typical AFL patients, the AF group did still show a significant improvement in HRQOL at one year. Moreover, the post-ablation rate of AF appears to be time-dependent with previous figures for incidence of AF during three-year follow-up varying widely, from 15% [21] to 82% [22]. This suggests that typical-AFL populations differ with respect to incidence of AF, and raises the question of how CTI ablation in AFL can be so effective within a context of such a frequent secondary arrhythmia. Willems et al have found an improvement in quality of life in a cohort of patients undergoing CTI ablation for typical AFL and with an AF incidence of 60% at 2 years [23].

Undoubtedly, the explanation will ultimately be found to be complex, but the following factors should be considered.

First, AF is better tolerated than AFL. AFL normally presents with a conduction ratio of 2:1 or 3:1, producing heart rates between 150 lpm and 130 lpm. The fact that it is also more difficult to achieve adequate pharmacological control of heart rate in AFL than in AF could explain why symptoms in AFL patients are generally more severe. In a cohort of patients with AF being treated with anti-arrhythmic drugs the presence of AFL during follow-up was associated with a lower rate of return to sinus rhythm due to anti-arrhythmic drugs and with a higher number of hospitalisations than AF recurrence. In the same population there was an 8.5% incidence of AFL having a 1:1 conduction ratio, a highly symptomatic form of AFL [24]. Therefore, AFL appears to be an arrhythmia with a higher impact on HRQOL than that of AF because it is more symptomatic, more refractory to pharmacological treatment – in terms of control of both heart rate and rhythm – and leads to a greater number of hospitalisations than AF.

Second, reduction of number of symptomatic palpitation episodes and AF burden following ablation for AFL. A reduction in the number of AF episodes post-ablation has been widely reported [16,25,26]. One explanation could be the elimination of AFL as a trigger of AF events. It has also been suggested that normalisation of the effective refractory period and inverse electrical remodelling in the atrium reduce the frequency of AF [27]. In our patient cohort, the recorded incidence of AF at one-year follow-up was reduced from 43% basal to 25%. This reduction should, however, be interpreted with caution as AF diagnosis was based on symptoms and events registered by Holter monitoring over the one-year period. A more rigorous approach might be to monitor and diagnose oligosymptomatic episodes, and/or to have a longer follow-up period in which to gather data on the development of AF in post-ablation patients. Ellis et al [22] have reported an incidence in post-ablation AF of up to 82% at 3 years and 3 months. Similarly, Chinitz et al [28] have reported an occurence of AF of 50% at a follow-up of 2.5 years in a cohort of patients with typical isolated AFL undergoing CTI ablation. More recently, a recurrence of AF has been reported in 90% of patients receiving hybrid treatment (ablation for AFL + antiarrhythmic treatment for AF) at 5-year follow-up [29]. It seems likely that AFL and AF constitute different electrocardiolographic expressions of a common atrial disease and that CTI ablation does not eliminate the risk of subsequent AF [30].

Patients who had experienced only a single AFL event showed a similar improvement in HRQOL to those with recurrent AFL, although the latter group showed a tendency towards increased improvement in the physical dimensions. The benefit of CTI ablation over amiodarone treatment in patients who had experienced only a single episode of AFL had been described previously [31]. This datum is important because patients with a single AFL event are usually treated more conservatively, on the assumption that they will benefit less from ablation therapy and recurrence will be delayed.

No differences in HRQOL were seen with respect to ventricular cycle length of ≤500 ms or >500 ms. Neither were any differences seen in relation to presence or absence of tachycardiomyopathy, which makes sense in the absence of ventricular-cycle-related differences. Our observation that patients who had presented with persistent AFL and a ventricular cycle of ≤500 ms (heart rate ≥120 lpm) showed a significant improvement in four HRQOL dimensions (compared with patients with persistent AFL and ventricular cycle of >500 ms), however, demonstrates that when AFL is persistent ventricular cycle length can affect HRQOL.

Analysing differences in HRQOL with respect to combinations of characteristics (i. persistent AFL + ventricular cycle ≤500 ms; ii. structural heart disease + ventricular cycle ≤500 ms + recurrent AFL) revealed greater differences, particularly in relation to physical dimensions. While these differences are potentially important, the fact that the number of cases in each group was low would have reduced the capacity for statistical tests to show significant differences in other dimensions. Indeed, it seems possible that the combination of structural heart disease, ventricular cycle ≤500 ms and recurrent AFL will be a better predictor of improvement in HRQOL scores following CTI ablation than will each of those factors individually.

### Limitations

The majority of studies using the SF-36 questionnaire have observed ceiling effect (more than 15% of patients had the maximum value for a dimension) on the dimensions of Physical Role and Emotional Role. In our cohort we have observed a ceiling effect in the basal dimensions Physical Role, Bodily Pain, Social Function and Emotional Role and at one-year follow-up in the same dimensions and additionally in Physical Function. Therefore the presence of a ceiling effect might have caused an underestimation of the size of change in HRQOL over the one-year follow-up period. In three dimensions (General Health, Vitality and Mental Health) a ceiling effect was not seen, and thus the values obtained in those dimensions were particularly valuable in assessing improvement in HRQOL at one year.

Other limitations were the following. a.) Because of the advanced age and comorbidities in our population, we lost some patients during follow-up due to death or incapacity to complete the second questionnaire at one year. b.) The low number of patients we recruited. c.) The observational approach we used. Because we did not randomly assign patients to treatment and placebo groups, we could not make inferences about cause and effect. It will therefore be important to carry out controlled studies in the future. d.) We used only a general type of questionnaire to evaluate quality of life, and not one designed for the context of cardiac arrhythmias [32,33] and e) A 7-day Holter recording is a rather limited method to assess AF burden. An implantable loop recorder would have provide more accuracy [34].

## Conclusions

In conclusion, the main finding of our analysis of changes in HRQOL scores of typical AFL patients one year after CTI ablation treatment, in relation to age, sex and several relevant cardiovascular parameters, was that improvement in HRQOL was most strongly associated with absence of AF. We also found the presence of structural cardiopathy to be independently associated with a greater improvement in in the physical summary component.

## Abbreviations

AFL, Atrial flutter; AF, Atrial fibrillation; HRQOL, Heart -related quality of life; CTI, Cavotricuspid Isthmus; SF-36, Short-form 36.

## Competing interests

There are no conflict of interest for any of the authors for this manuscript.

## Authors’ contributions

JG was responsible for the conceptuation and design of the study, performing electrophysiological procedures, acquisition of data, draft and revision of the manuscript. FG: participated in design, statistical analyses and interpretation of data and in manuscript revision, PC was involved in acquisition of data, consent inform and Ethical Committee relationships and clinical follow-up. JM and XF performed electrophysiological procedures and clinical follow-up. CM and AH participated in conception and design of the study and finally JGJ gave the final approval of the version to be published. All authors read and approved the final manuscript.

## References

[B1] Saoudi N Cosío F Waldo A Chen S Iesaka Y Lesh M Saksena S Working group of Arrhythmias of the European of Cardiology and the North American Society of Pacing and Electrophysiology. A classification of atrial flutter and regular atrial tachycardia according to electrophysiological mechanisms and anatomical bases Eur Heart J 2001 22 1162 1182 10.1053/euhj.2001.2658 11440490

[B2] García-Cosío F Pastor A Núñez A Magalhaes A Awamleh P Flúter auricular: perspectiva clínica actual Rev Esp Cardiol 2006 59 816 831 10.1157/13091886 16938231

[B3] Feld GK Fleck RP Chen PS Boyce K Bahnson TD Stein JB Calisi CM Radiofrequency catheter ablation for the treatment of human type I atrial flutter. Identification of a critical zone in the reentrant circuit by endocardial mapping techniques Circulation 1992 86 1233 1240 10.1161/01.CIR.86.4.1233 1394929

[B4] Cosío FG Arribas F López-Gil M Palacios J Atrial flutter mapping and ablation. I. Atrial flutter mapping Pacing Clin Electrophysiol 1996 19 841 853 10.1111/j.1540-8159.1996.tb03368.x 8734753

[B5] Calkins H Canby R Weiss R Taylor G Wells P Chinitz L Milstein S 100 W Atakr II Investigator Group. Results of catheter ablation of typical atrial flutter Am J Cardiol 2004 94 437 442 10.1016/j.amjcard.2004.04.058 15325925

[B6] Feld GK Wharton M Pluma V Daoud E Friehling T Epstein L for the EPT-100 XP Cardiac Ablation System Investigators. Radiofrequency catheter ablation of type 1 atrial flutter using large-tip 8 - or 10-mm electrode catheters and a high – output radiofrequency energy generator. Results of a multicenter safety and efficacy study J Am Coll Cardiol 2004 43 1466 1472 10.1016/j.jacc.2003.11.036 15093885

[B7] O´Callaghan PA Meara M Kongsgaard E Poloniecki J Luddington L Foran J Camm AJ Symptomatic improvement after radiofrequency catheter ablation for typical atrial flutter Heart 2001 86 167 171 10.1136/heart.86.2.167 11454833 PMC1729856

[B8] Lee SH Tai CT Yu WC Chen YJ Hsieh MH Tsai CF Chang MS Effects of radiofrequency catheter ablation on quality of life in patients with atrial flutter Am J Cardiol 1999 84 278 283 10.1016/S0002-9149(99)00276-3 10496435

[B9] García Seara J Gude F Cabanas P Martínez Sande JL Fernández López X Elices J Brugada Terradellas J Quality of life differences in patients with typical atrial flutter following cavotricuspid isthmus ablation Rev Esp Cardiol 2011 64 401 408 10.1016/j.recesp.2010.12.014 21482002

[B10] Saoudi N Ricard P Rinaldi JP Yaïcil K Darmon JP Anselme F Methods to determine bidirectional block of the cavotricuspid isthmus in radiofrequency ablation of typical atrial flutter J Cardiovasc Electrophysiol 2005 16 801 803 10.1111/j.1540-8167.2005.40624.x 16050843

[B11] Shah D Haïssaguerre M Takahashi A Jaïs P Hocini M Clementy J Differential pacing for distinguishing block from persistent conduction through an ablation line Circulation 2000 102 1517 1522 10.1161/01.CIR.102.13.1517 11004142

[B12] Ware JE Jr Snow KK Kosinski M Gandek B Scoring the SF-36 Ware JE Jr. SF-36 Health Survey. Manual & Interpretation Guide 1997 2 The Health Institute, New England Medical Center, Boston 6:1 6:22 6

[B13] Vilagut G Ferrer M Rajmil L Rebollo P Permanyer G Quintana J Santed R El cuestionario de salud SF-36 español: una década de experiencia y nuevos desarrollos Gac Sanit 2005 19 135 150 10.1157/13074369 15860162

[B14] Vilagut G Valderas JM Ferrer M Garin O López-García E Alonso J Interpretación de los cuestionarios SF-36 y SF-12 en España: componentes físico y mental Med Clin 2008 130 726 735 10.1157/13121076 18570798

[B15] Gilligan D Zakaib J Fuller I Shepard R Dan D Wood M Long-term outcome of patients after successful radiofrequency ablation for typical atrial flutter Pacing Clin Electrophysiol 2003 26 Pte I 53 58 12685140 10.1046/j.1460-9592.2003.00150.x

[B16] Schmieder S Ndrepepa G Dong J Zrenner B Schreieck J Schneider AE Karch M Acute and long-term results of radiofrequency ablation of common atrial flutter and the influence of the right atrial isthmus ablation on the ocurrence of atrial fibrillation Eur Heart J 2003 24 956 962 10.1016/S0195-668X(02)00846-1 12714027

[B17] Cuesta A Mont L Alvarenga N Rogel U Brugada J Comparison of 8-mm-tip and irrigated-tip catheters in the ablation of isthmus-dependent atrial flutter: a prospective randomized trial Rev Esp Cardiol 2009 62 750 756 10.1016/S0300-8932(09)71688-4 19709510

[B18] Chen J De Chillou C Ohm OJ Hoff PI Rossvoll O Andronache M Sadoul N Acute resumption of conduction in the cavotricuspid isthmus after catheter ablation in patients with common atrial flutter Europace 2002 4 255 263 10.1053/eupc.2002.0243 12134971

[B19] Scavée C Georger F Jamart J Mancini I Collet B Blommaert D De Roy L Is a cooled tip catheter the solution for the ablation of the cavotricuspid isthmus? Pacing Clin Electrophysiol 2003 26 328 331 10.1046/j.1460-9592.2003.00043.x 12687839

[B20] Anselme F Saoudi N Poty H Douillet Cribier A Radiofrequency catheter ablation of common atrial flutter: significance of palpitations and quality of life evaluation in patients with proven isthmus block Circulation 1999 99 534 540 10.1161/01.CIR.99.4.534 9927400

[B21] Da Costa A Romeyer-Bouchard C Zarqane-Sliman N Messier M Samuel B Kihel A Faure E Impact of first line radiofrequency ablation in patients with lone atrial flutter on the long term risk of subsequent atrial fibrillation Heart 2005 91 97 98 10.1136/hrt.2003.033308 15604348 PMC1768657

[B22] Ellis K Wazni O Marrouche N Martin D Gillinov M McCarthy P Saad E Incidence of atrial fibrillation post-cavotricuspid isthmus ablation in patients with typical atrial flutter J Cardiovasc Electrophysiol 2007 18 799 802 10.1111/j.1540-8167.2007.00885.x 17593230

[B23] Anné W Willems R Adriaenssens B Admas J Ector H Heidbüchel H Long-term symptomatic benefit after radiofrequency catheter ablation for atrial flutter despite a high incidence of post-procedural atrial fibrillation Acta Cardiol 2006 61 75 82 10.2143/AC.61.1.2005143 16485736

[B24] Riva S Tondo C Carbucicchio C Galimberti P Fassini G Della Bella P Incidence and clinical significance of transformation of atrial fibrillation to atrial flutter in patients undergoing long-term antiarrhythmic drug treatment Europace 1999 1 242 247 10.1053/eupc.1999.0048 11220561

[B25] Bertaglia E Zoppo F Bonso A Proclemer A Verlato R Corò L Mantovan R Long term follow up of radiofrequency catheter ablation of atrial flutter: clinical course and predictors of atrial fibrillation occurrence Heart 2004 90 59 63 10.1136/heart.90.1.59 14676244 PMC1768035

[B26] Pérez F Schubert C Parvez B Pathak V Ellenbogen KA Wood MA Long-term outcomes after catheter ablation of cavo-tricuspid isthmus dependent atrial flutter: A Meta-Analysis Circ Arrhythm Electrophysiol 2009 2 393 401 10.1161/CIRCEP.109.871665 19808495

[B27] Meissner A Christ M Maagh P Borchard R Bracht MV Wickenbrock I Trappe Quality of life and occurrence of atrial fibrillation in long-term follow-up of common type atrial flutter ablation: Ablation with irrigated 5 mm tip and convencional 8 mm tip electrodes Clin Res Cardiol 2007 96 1 9 10.1007/s00392-006-0447-y 17721735

[B28] Chinitz JS Gerstenfeld EP Marchilinski FE Callans DJ Atrial fibrillation is common after ablation of isolated atrial flutter during long-term follow-up Hear Rhythm 2007 4 1029 1033 10.1016/j.hrthm.2007.04.002 17675077

[B29] Anastasio N Frankel D Deyell M Zado E Gerstenfeld E Dixit S Cooper J Nearly uniform failure of atrial flutter ablation and continuation of antiarrhythmic agents (hybrid therapy) for the long-term control of atrial fibrillation J Interv Card Electrophysiol 2012 10.1007/s10840-012-9679-0 22552760

[B30] Laurent V Fauchier L Pierre B Grimard C Babuty D Incidence and predictive factors of atrial fibrillation after ablation of typical atrial flutter J Interv Card Electrophysiol 2009 24 119 125 10.1007/s10840-008-9323-1 18982436

[B31] Da Costa A Thêvenin J Roche F Romeyer C Abdellaoui L Messier M Denis L Results from the Loire-Ardèche-Drôme-Isère-Puy-de-Dôme (LADIP) trial on atrial flutter, a multicentric prospective randomized study comparing amiodarone and radiofrequency ablation after the first episode of symptomatic atrial flutter Circulation 2006 Oct 17 114 16 1676 1681 Epub 2006 Oct 9 10.1161/CIRCULATIONAHA.106.638395 17030680

[B32] Härden M Nyström B Kulich K Carlsson J Bengtson A Edvardsson N Validity and reliability of a new, short symptom rating scale in patients with persistent atrial fibrillation Health Qual Life Outcomes 2009 7 65 10.1186/1477-7525-7-65 19604399 PMC2717073

[B33] Badia X Arribas F Ormaetxe J Peinado R Sainz de los Terreros M Development of a questionnaire to measure health-related quality of life (HRQoL) in patients with atrial fibrillation (AF-QoL) Health Qual Life Outcomes 2007 5 37 10.1186/1477-7525-5-37 17610734 PMC1936414

[B34] Eitel C Husser D Hindricks G Frühauf M Hilbert S Arya A Performance of an implantable automatic atrial fibrillation detection device: impact of software adjustments and relevance of manual episode analysis Europace 2011 13 480 485 10.1093/europace/euq511 21325346 PMC3065917

